# One-year spatiotemporal database of Emergency Medical Service (EMS) calls in Mashhad, Iran: data on 224,355 EMS calls

**DOI:** 10.1186/s13104-022-05905-8

**Published:** 2022-01-25

**Authors:** Soheil Hashtarkhani, Behzad Kiani, Alireza Mohammadi, Shahab MohammadEbrahimi, Saeid Eslami, Mahmood Tara, Stephen A. Matthews

**Affiliations:** 1 Department of Medical Informatics, School of Medicine, Mashhad University of Medical Sciences, Mashhad, Iran; 2 Department of Geography and Urban Planning, Faculty of Social Sciences, University of Mohaghegh Ardabili, Ardabil, Iran; 3 Department of Sociology and Criminology, and Department of Anthropology, The Pennsylvania State University, University Park, PA USA

**Keywords:** Emergency Medical Services, EMS dataset, Spatial analysis, Mashhad, Iran

## Abstract

**Objectives:**

Emergency Medical Services (EMS) is the first point of service for the people who are in critical condition and in need of urgent health care. In Iran, as in other countries, people in need of emergency services often die or are left with a permanent injury due to the poor EMS-related infrastructure. It has been shown that a detailed examination of the response times and the spatiotemporal pattern of EMS calls for service can lead to improvements in time-sensitive patient outcomes. We performed a spatiotemporal study in city of Mashhad, the second-most populous city of Iran, to investigate the pattern of the EMS calls and now wish to release a comprehensive dataset resulting from this study.

**Data description:**

The data include three data files plus a data dictionary file. Data file 1 contains the characteristics of EMS requests including sex, age group, date of call, different time periods of each EMS missions, the census tracts’ ID of callers, the chief complaint, and the EMS mission result. Two spatial data files include the boundaries of the census tracts in Mashhad and the point location of all EMS stations, respectively. A data dictionary file defines all fields and values across the data files.

## Objective

Given the vital role of high-quality and efficient emergency care in saving patients’ lives from unpredicted life-threatening injuries or critical conditions, the Emergency Medical Service (EMS) goal is to concentrate on providing timely and well-monitored care [1, 2]. To achieve this goal, it is crucial to have a deeper understanding of EMS system including the demographic and geographical characteristics of the callers and distribution of EMS infrastructure resources [3]. In this regard, Geographical Information Systems (GISs) is a highly useful toolbox for decision making and it has capabilities to visualize space-time information [4] Since adjacent areas have similar characteristics in terms of spatial and temporal aspects, applying GIS to discover patterns of health events has received more attention recently [5, 6].

Mashhad is the capital city of Razavi Khorasan Province with an estimated population of 3,800,000 inhabitants [7]. Mashhad also is the most popular tourist destination in the country, with over 20+ million visitors per annum [8]. A central dispatch center in the city of Mashhad manages all EMS requests of Mashhad County. Spatio-temporal patterns of EMS requests were investigated using spatio-temporal scan statistics approach in Mashhad [9]. Here, a comprehensive dataset related to this study is offered for further investigation to identify clustering patterns and potential geographic characteristics of EMS requests in a densely populated city.

## Data description

Table 1 shows the details of the three data files plus the data dictionary file. Data on 224,355 ambulance calls from 1st of Jun 2018, to 31st of May 2019 were obtained from the database of Mashhad EMS centre (Data file 1). This dataset contains 12 fields including: (a) date of call, (b) patient sex [male (M), female (F), or Not Available (N/A)], (c) patient age (categorized in 10-year intervals), (d) location (inside or outside the city), (e) census tract ID (1 to 1301 for calls in urban area), (f) primary or chief medical complaint (in 12 categories), (g) EMS mission result (in five categories), (h) call time (the time EMS receives the call), (i) start time of the mission, (j) time of arrival at the scene by EMS, (k) time of starting the transfer from the scene to a hospital, and (l) time of arrival at the hospital. Notably, the location of callers was recorded by latitude and longitude in the EMS system. Altered level of consciousness, trauma injuries and cardiovascular incidents are the main reasons of emergency requests in Mashhad [9]. In order to protect the privacy of callers, the point location was linked to a larger area, a census tract, using the spatial join tool of ArcGIS v. 10.6.

**Table 1:** Overview of the data files

Label	Name of data file/data set	File types (file extension)	Data repository and identifier (DOI or accession number)
Data file 1	Mashhad_EMS_Calls	MS Excel (.xlsx)	Harvard Dataverse (https://doi.org/10.7910/DVN/JZEVI8) [10]
Data file 2	Mashhad_Census_Tracts	Shape file (.shp)	Harvard Dataverse (https://doi.org/10.7910/DVN/JZEVI8) [10]
Data file 3	Mashhad_Ambulance_Stations	Shape file (.shp)	Harvard Dataverse (https://doi.org/10.7910/DVN/JZEVI8) [10]
Data dictionary	Mashhad_EMS_Help	MS Excel (.xlsx)	Harvard Dataverse (https://doi.org/10.7910/DVN/JZEVI8) [10]

Census tracts’ boundaries and their population were obtained from municipality of Mashhad (Data file 2). Data for the locations of 53 ambulance stations in urban area as well as 13 ambulance stations in the surrounding countryside were obtained from EMS dispatch centre of Mashhad (Data file 3). Finally, following another data note [11], a data dictionary document is included that provides definitions of all variables and values used in the three data files.

The datasets provided in this study can be used by researchers in different disciplines such as health geography, urban planning, public health and health disparities. Descriptive thematic maps can be produced to highlight the distribution of EMS requests during the different time intervals (by time of day, day, week or month).

Spatial clustering techniques like local Moran’s I [12] or Getis-Ord Gi* [13] can be applied to this data to identify high-demand or low-demand clusters of emergency services [14]. High-demand clusters (hot spots) identify the areas having significantly greater EMS requests than expected while low-demand clusters reveal the regions with a significantly lower EMS request rate (cold spots) [15]. Spatiotemporal clustering techniques like space–time scan statistics [16] can be applied to discover high-risk areas in different time intervals (hour, day, week, month). These analytical approaches can help policymakers effectively allocate resources across the city and based on different temporal intervals. Also, these techniques can be applied separately for specific primary or chief medical complaints such as trauma or cardiovascular diseases. In addition to spatiotemporal analyses, these data can be linked to contextual factors for different geographically defined areas—contextual factors such as socio-economic variables (e.g., socioeconomic status) or environmental variables (e.g., air pollution)—to further investigate the impact of contextual factors on EMS requests. The research outputs can inform health policymakers and urban planners to develop appropriate strategies to provide efficient and timely pre-hospital care services.

## Limitations

Mashhad is a dynamic city with millions of visitors per annum. There are many religious festivals across the year but the spatiotemporal patterning of visitors across census tracts in the city during the study period is not known. We do not know if a patient receiving EMS care is a resident of Mashhad or a tourist. While we can use recent population totals for each census tract the true population at risk is not known. As with all EMS data there are issues of quality. While some data are automated (e.g., dispatch time, arrival time) EMS staff are working in emergency and high-stress situations and specific times of treatment at the service site may have varying levels of temporal precision. There is no valid data for visitors in Mashhad. However, most of the visitors come to the city in summers.

## Data Availability

The data described in this Data note can be freely and openly accessed on the Harvard Dataverse under (https://doi.org/10.7910/DVN/JZEVI8) [10]. Please see Table 1 and the reference list for details and links to the data.

## Abbreviations

EMS: Emergency Medical Services
GIS: Geographical information system
